# Orientational Order of Phenyl Rotors on Triangular Platforms on Ag and Au(111)

**DOI:** 10.1021/acsnano.5c14953

**Published:** 2025-10-27

**Authors:** Behzad Mortezapour, Sebastian Hamer, Rainer Herges, Roberto Robles, Richard Berndt

**Affiliations:** † Institut für Experimentelle und Angewandte Physik, 234685 Christian-Albrechts-Universität zu Kiel, 24098 Kiel, Germany; ‡ Otto-Diels-Institut für Organische Chemie, Christian-Albrechts-Universität zu Kiel, 24098 Kiel, Germany; § 9179 Centro de Física de Materiales CFM/MPC (CSIC-UPV/EHU), 20018 Donostia-San Sebastián, Spain

**Keywords:** self-assembly, van der Waals interaction, dimerization, trioxatriangulenium, Ag(111), scanning tunneling microscopy

## Abstract

We investigated trioxatriangulenium functionalized with phenyl (phenyl-TOTA) on the (111) surfaces of Ag and Au using low-temperature scanning tunneling microscopy (STM) and density functional theory (DFT). On Ag(111), the molecules form hexagonal arrays, and on Au(111), honeycomb patterns are also observed. The orientations of the phenyl moieties are resolved on both substrates. On Ag(111), the orientations are parallel within a row and they differ by approximately 60° between adjacent molecular rows, and STM images suggest dimerization of the molecules. DFT calculations for Ag(111) reveal that van der Waals interactions dominate this system. The optimized structure matches the experimental pattern, and the simulated STM images exhibit apparent dimerization. The dimerization results from an asymmetry of the phenyl wave function, which reflects intramolecular hydrogen bonding between the ligand and an oxygen atom within the triangulenium platform. The orientation of the phenyl moieties is explained by the interaction of each phenyl subunit with its triangulenium platform combined with the direct long-range interaction between phenyl moieties across molecules.

## Graphical abstract

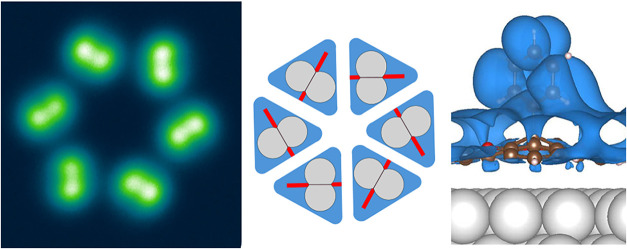

The adsorption of molecules onto single-crystalline surfaces in vacuum or at liquid–solid interfaces often results in the self-assembly of decorative and potentially useful two-dimensional patterns.^1–3^ Understanding the patterns, which depend on various molecule–molecule and molecule–substrate interactions, as well as the detailed parameters of the preparation, is a formidable task. Platforms in the shape of an equilateral triangle, deposited on a hexagonally symmetric substrate may seem to be a comparatively simple case. Indeed, various networks with honeycomb or hexagonal symmetries have often been observed.^4–11^ To add functionality to these arrays, molecules that serve as a platform for various ligands have been used. In particular, the triangular compound trioxatriangulenium (TOTA) (inset of Figure 1(a)) forms hexagonal or honeycomb arrays on the (111) surfaces of Ag and Au and can easily be functionalized with various axial ligands that stand vertically on the TOTA plane.^12–18^ The functional subunits in these arrays are separated by a distance of 1.01 to 1.04 nm.[Fn fn1]

**Figure 1:**
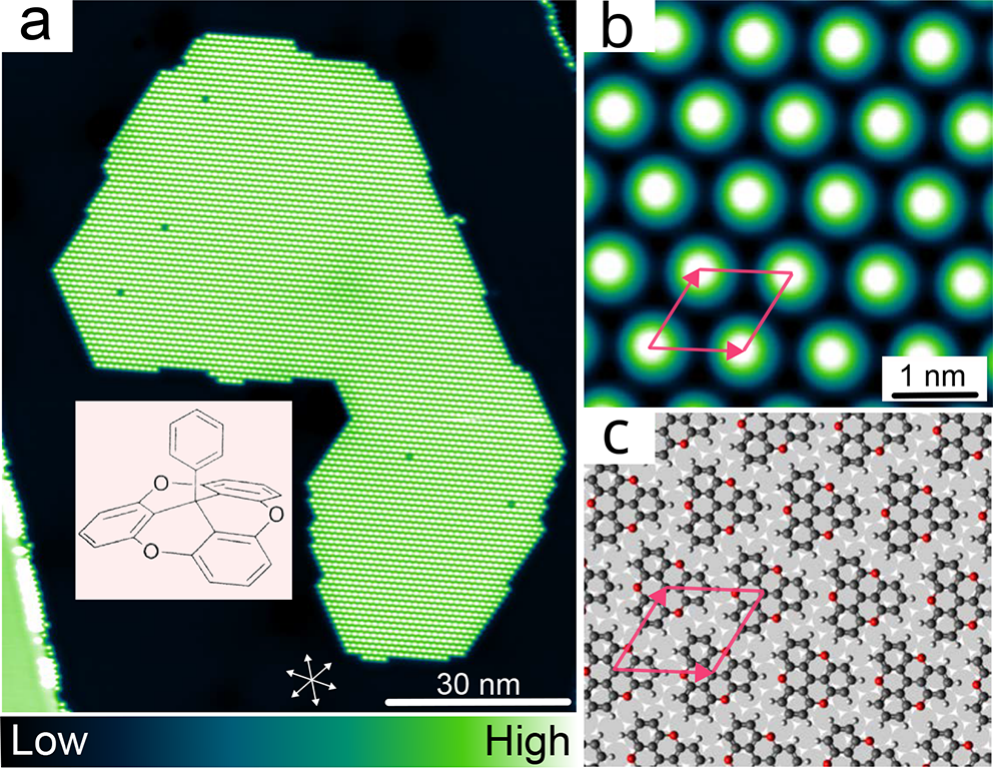
(a) Overview topograph (*V* = 0.15 V) of a submonolayer amount of phenyl-TOTA on Ag(111). The inset shows a scheme of phenyl-TOTA. (b) More detailed topograph (*V* = 0.7 V) from the interior of a molecular island. A unit cell is indicated. (c) Model of the molecular layer and the substrate as determined from experimental STM images.

Phenyl-TOTA, which we investigate here, is particularly interesting because interactions between aromatic molecules are relevant in many areas of chemistry and engineering.^19^ The model case of benzene–benzene interaction has been computationally studied at various theoretical levels.^19–24^ While the detailed geometric arrangement of the molecules is known to play a role, the interaction energy at nm distance is expected to be of the order of few meV.^24^

In the present case, however, the functionalization with a phenyl subunit has a striking effect. Using scanning tunneling microscopy (STM), the orientation of the phenyl moiety is resolved. We find a striped phase of alternating rows that select two of three phenyl orientations differing by ≈60°. In addition, the STM images suggest a dimer-like pairing of the molecules. Density functional theory (DFT) calculations including van der Waals corrections, reproduce several important experimental findings and lead to the following interpretation: the van der Waals interaction is by far the strongest interaction in this system. In fact, it decreases the distance between the molecules and the substrate, and governs the orientation of the phenyls. The calculated lowest energy structure matches the experimental pattern and leads to apparent dimerization in STM images. This dimerization is due to the distortion of the phenyl wave function that results from the intramolecular hydrogen bonding between the ligand and an oxygen atom within the triangulenium platform. The regular pattern of phenyl orientations results from the combination of two effects: the intramolecular hydrogen bonding, which determines three possible orientations of each ligand on the triangulenium platform, and the direct long-range interactions between the ligands, which determines their relative orientation.

## Results and Discussion

### Experimental Results on Ag(111)

#### Molecular Pattern on Ag(111)

Figure 1(a) presents an overview of a 92 × 150 nm^2^ area of a Ag(111) surface that is covered with a submonolayer amount of phenyl-TOTA. The area exhibits a wide terrace and two substrate steps, which run nearly vertically through the image. The steps are covered by strings of molecules, both on the upper and the lower adjacent terrace. However, there seems to be no clear ordering within these strings. On the wide terrace, however, a well-ordered molecular island is observed. The individual molecules are arranged in a hexagonal pattern and the island edges are preferentially oriented along densely packed directions of the molecular pattern. The defect density in the island is very low (5 depressions among approximately 4700 molecules). The large size and high degree of order of the island indicates that the regularity of the molecular arrangement leads to a significant energy gain.

Closer inspection of areas within a molecular island (Figure 1(b)) reveals a hexagonal lattice of nearly circular protrusions. Using atomically resolved images of the Ag substrate for calibration, we determined the nearest-neighbor distance to be 1.04 ± 0.01 nm and the angle between the close-packed directions of the substrate and the superlayer to be ±13° ± 2°. The centers of the molecules are located above 3-fold hollow sites of the Ag(111) lattice.

The experimental images suggest the model shown in Figure 1(c). In this model the phenyl-TOTA molecules arranged in a corner-to-side fashion that enabled six hydrogen bonds between the TOTA platform and its six nearest neighbors. This model is identical to the earlier proposed model for methyl-TOTA on Ag(111).^25^ More precisely, methyl-TOTA forms two distinct patterns. At low molecular densities, a honeycomb lattice was found, with triangular TOTA platforms arranged side-by-side. This configuration enables two hydrogen bonds between adjacent molecules. At high densities, the pattern is identical to the one observed here, with adjacent molecules arranged in a corner-to-side fashion, forming one hydrogen bond per neighbor.

#### Spectroscopic Effects on Ag(111)

STM images of the molecular layer at negative *V* do not show a pronounced voltage dependence, except for a gradual change in the apparent height of the island relative to the substrate. This is consistent with the fairly featureless spectra of the differential conductance d*I*/d*V* (not shown). However, at *V* > 0 (Figure 2), the molecular pattern evolves in an intriguing fashion. While each molecule gives rise to a nearly circular protrusion at low bias (panel a), an elliptical shape becomes discernible near 1.9 V. At 2.1 and 2.2 V (panels b and c) the ellipses are clearly visible. We find that the orientation of the long axes changes by ≈60° between adjacent densely packed rows. A further increase of *V* to 2.4 V adds new image features. In particular, the ellipses exhibit a central constriction, which may indicate a poorly resolved nodal line. The changes in the images within this voltage range are reflected by a peak in d*I*/d*V* (Figure 3), which suggests that the lowest unoccupied molecular orbital (LUMO) begins to dominate the image contrast. As will be discussed below, the elliptical shape can be explained from the local density of states of the π-orbital that is located around the plane of the phenyl subunit. Therefore, the STM images reveal a unit cell with two molecules that differ by the orientation of the phenyls.

**Figure 2:**
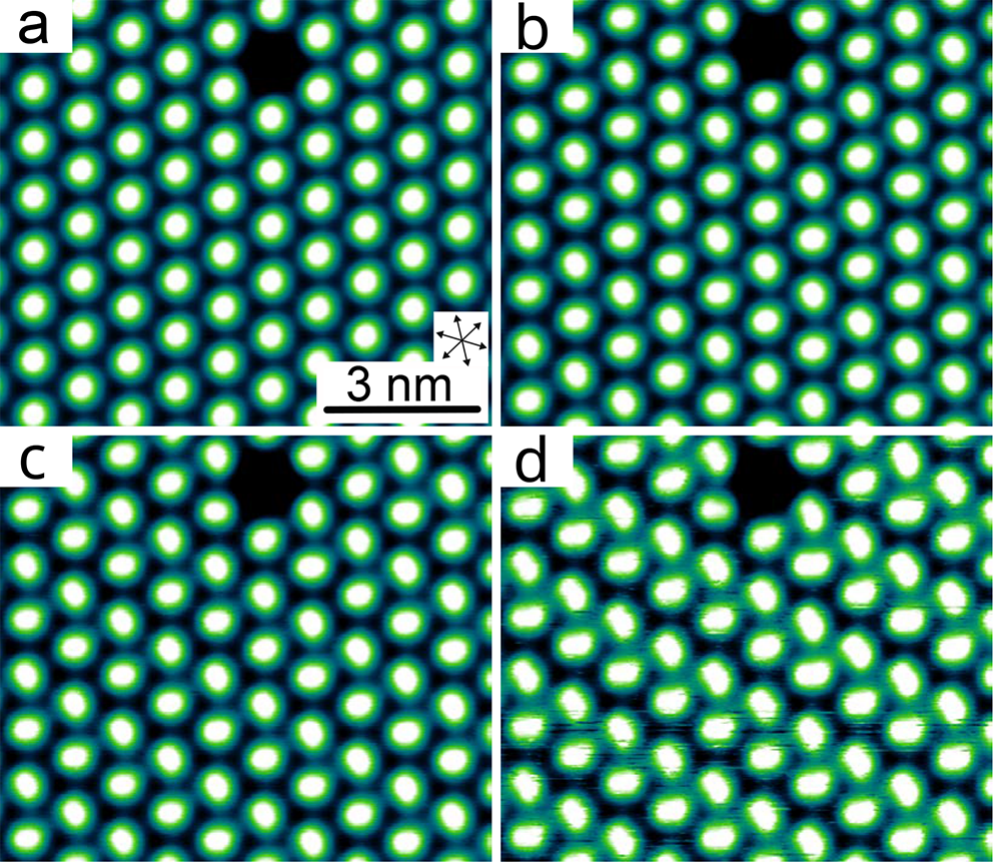
Topographs of a molecular island recorded at different sample voltages *V*. A defect (most likely a TOTA platform without phenyl ligand) near to top of the image serves as a marker. (a) At *V* = 0.3 V, the molecules appear as nearly circular protrusions. (b, c) As *V* is increased to 2.1 and 2.2 V, the molecules develop an increasingly elliptical shape. The long axes of the ellipses exhibit alternating orientations. (d) At *V* = 2.4 V, the orientations of the ellipses are clearly discernible. In addition, some indication of a constriction, possibly a nodal line, is barely visible. Moreover, the dense molecular rows appear to have rearranged into double rows that are separated by dark grooves.

**Figure 3:**
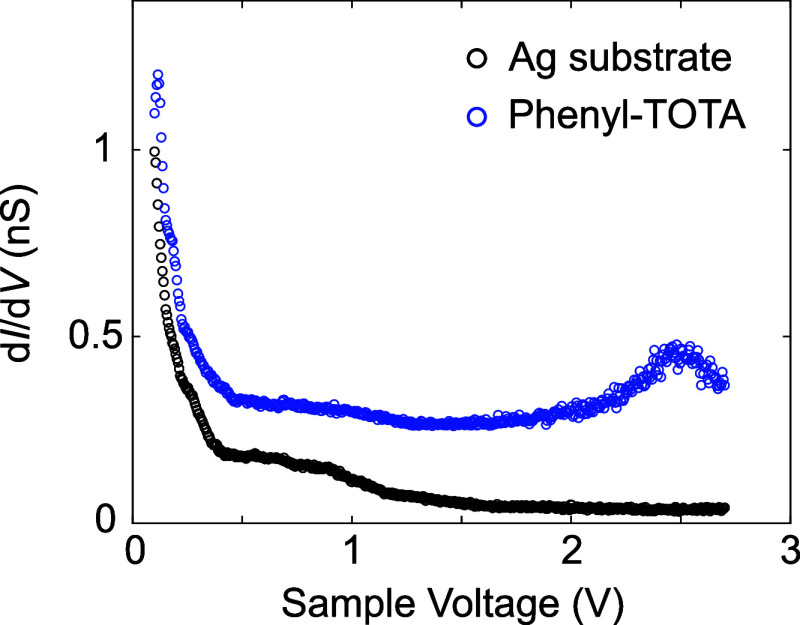
Spectra of the differential conductance d*I*/d*V*, recorded at constant current. While the spectrum of the Ag(111) substrate (black) is fairly featureless at voltages exceeding 1 V, the spectrum of phenyl-TOTA (blue) exhibits a clear resonance centered near 2.5 V. The blue curve has been arbitrarily shifted for clarity. It should be noted that features in constant-current d*I*/d*V* spectra are slightly shifted toward the Fermi level compared to constant-height data.^26^

Assuming that the image contrast above the phenyl reflects the π orbital, the orientation of the phenyl subunits may be inferred. Figure 4 shows a detailed image of the elliptical image contrasts and a corresponding model. It suggests that the phenyl subunits are parallel to each other along one direction of the unit cell and that they are rotated by 60° with respect to each other along the other direction.

**Figure 4:**
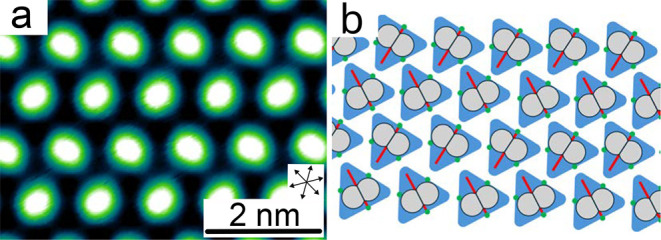
(a) Topograph (*V* = 2.2 V) showing the orientation of the molecular ellipses. (b) Model of the TOTA platforms and the phenyl orientation (red lines) along with schematic π orbitals (gray patches). Green dots indicate O atoms involved in bonding to the neighboring molecules.

At slightly elevated bias voltages (Figure 2c,d), we find that pairs of dense molecular rows exhibit dimerization. In this case, two closely spaced rows are separated from their neighbors by dark lines. These lines appear ≈50 pm lower that the maxima of the image at this voltage. When the voltage is increased further, the tunneling current becomes unstable, inducing drastic changes in the topographs at 2.6 V and above as verified by subsequent imaging at nonperturbing voltages (see Supporting Information).

Finally, we find that the onset of the Ag(111) surface state is shifted from −67 mV below the Fermi level to higher energies within the molecular islands (Figure 5).[Fn fn2] Inside the island, we find a box-shaped contribution to the density of states at positive bias. The minimum of the surface state band, which is determined from the midpoint of the rise, is shifted to ≈110 mV. As expected, the rise is broadened on molecules closer to the island rim.^29,30^

**Figure 5:**
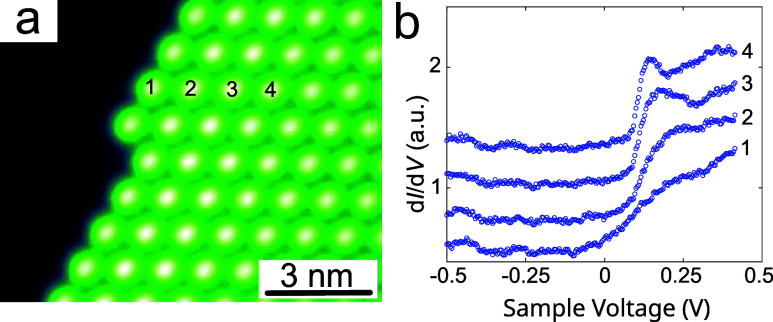
(a) Topograph of an island edge. The positions used for d*I*/d*V* spectroscopy are indicated by numbers. (b) Low-bias d*I*/d*V* spectra recorded from the four molecules marked in panel a. For clarity, the spectra have been arbitrarily shifted in the vertical direction. The current feedback was disabled at 30 pA and 0.5 V.

### Experimental Results on Au(111)

We performed a brief experimental study of phenyl-TOTA on Au(111). The herringbone reconstruction of this substrate apparently affects the pattern formation and leads to images as shown in Figure 6a. Most of the molecules are found in strings of hexagons in the fcc areas and at elbows of the reconstruction. At coverages approaching a monolayer, both hexagonally dense areas and honeycomb patterns were observed (not shown). A detailed image of an isolated hexagon is shown in Figure 6b. At the voltage used, the orientation of the phenyl subunit is hardly apparent as a nodal line. Imaging of the current at fixed tip height (Figure 6c) further confirms the data and reveals an angle of 60° between neighboring phenyl moieties, virtually identical to the observation made on Ag(111). We note that the phenyl pattern exhibits handedness and in fact both chiralities were observed from different hexagons. Interestingly, the intramolecular contrast is achieved at negative voltages. Indeed, d*I*/d*V* spectra (see Supporting Information) are featureless at positive voltages while a clear peak is observed near −1.9 V.

**Figure 6:**
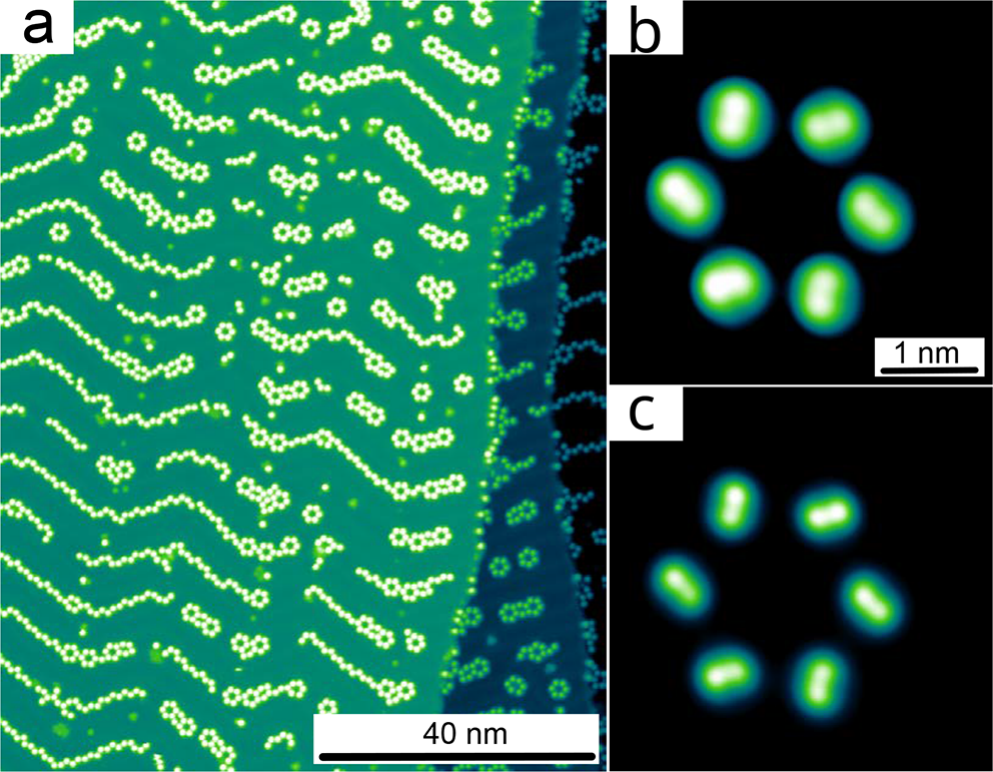
(a) Topograph (*V* = 0.5 V) of phenyl-TOTA on Au(111). (b) Topograph (*V* = −2, V) of an isolated hexagon of phenyl-TOTA molecules on Au(111). (c) Constant-height image of the same hexagon (*V* = −2 V).

### Theoretical Results

For DFT modeling, we focused on the Ag(111) substrate because the herringbone reconstruction of the Au(111) surface adds substantial complexity.

Using the experimentally determined unit cell containing two inequivalent molecules we have considered different ligand orientations (see Supporting Information). The most stable configuration is shown in Figure 7. As observed in the experimental data, the TOTA platforms are arranged in a hexagonal network. The phenyl ligands are oriented along a symmetry plane of the TOTA subunit that contains an O atom and the center of the platform. In this unit cell the phenyls are parallel to each other along the horizontal densely packed row direction. We find that they alternate between parallel rows. The third symmetry equivalent orientation is not present. These features reproduce the essential properties of the experimental structure data.

**Figure 7:**
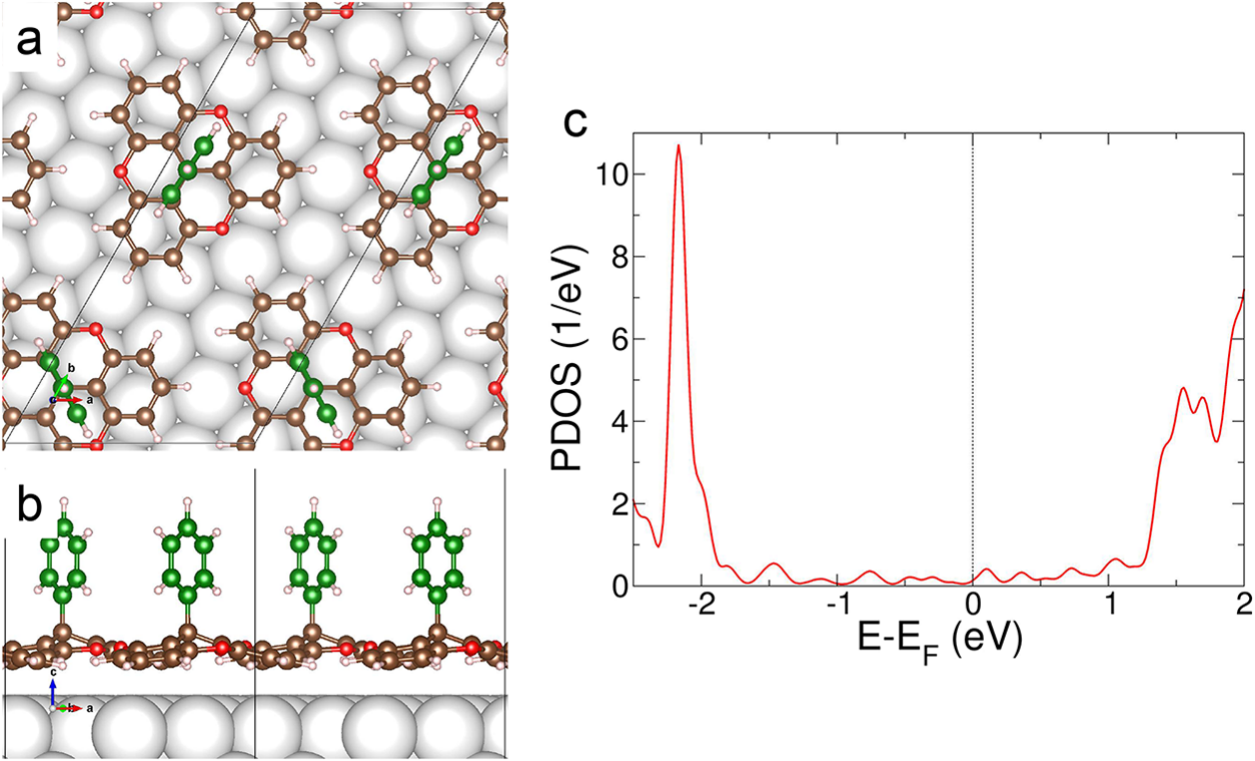
(a) Top and (b) side views of the most stable structure of a unit cell comprised of two phenyl-TOTA molecules on Ag(111). White, brown, red, and pink spheres represent Ag, C, O, and H atoms, respectively. Green spheres represent C atoms of the phenyl moiety. Black lines show the unit cell. (c) Density of states projected on the phenyl-TOTA molecule.

The adsorption energy of phenyl-TOTA on Ag(111) is *E*_ad_ = 2.238 eV per molecule. This energy can be decomposed in a vdW part *E*_ad,vdW_ = 2.584 eV and the rest, which is repulsive by *E*_ad,PBE_ = −0.346 eV. The reason for being repulsive is the strength of the vdW interaction, which decreases the distance between the molecular layer and the surface (from 3.4 Å without including vdW interactions in the calculation to 2.8 Å). The molecule adopts a position unfavorable for the PBE functional and the adsorption energy goes from *E*_ad,PBE_ = 0.082 to −0.346 eV, which is compensated by the vdW interaction which actually binds the molecule to the surface.

Next, we present constant-current images of phenyl-TOTA calculated for various voltages (Figure 8). While the molecules appear roundish at low bias, increased sample voltages lead first to an elliptical shape and finally to the appearance of a nodal plane that coincides with the phenyl plane. This contrast at positive bias is mainly due to the lowest unoccupied orbital (LUMO), which is antisymmetric with respect to the phenyl plane. Other orbitals with different symmetries only contribute at larger voltages. The agreement with the experimental images at comparable voltages in Figure 2 is quite good. Therefore, we conclude that the orientation of the phenyl subunits can be directly determined from those experimental images that resolve the nodal plane.

**Figure 8:**
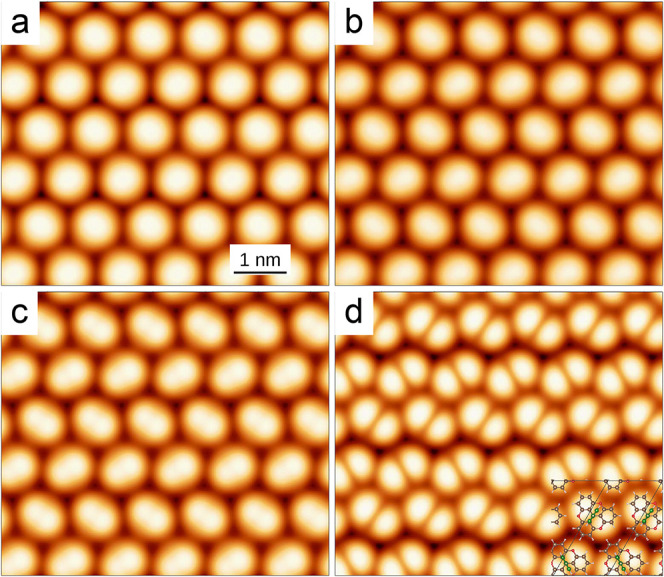
Calculated constant current STM images of the overlayer shown in Figure 7 for *V* = (a) 0.5, (b) 1.1, (c) 1.2, and (d) 1.5 V. The molecular structure is overlaid in the bottom right corner.

At large voltages the molecular rows seem to dimerize: the lower areas between the horizontal phenyl rows alternate between shallow and deep grooves (brighter and darker colors). This effect closely resembles the experimental observations. In order to analyze it, it is useful to inspect isodensity contours of the molecule (Figure 9). The symmetry of the phenyl subunit is reduced by the interaction of the lower hydrogen atoms with the oxygen and the opposing phenyl subunit of the platform. This effect is most clearly seen in the side view in Figure 9b, where the oxygen atom located on the right side of the image drastically deforms the electron cloud of the phenyl. As a result, the LUMO spills out further into the vacuum on the oxygen side. Taking into account the alternation of the phenyl orientations between parallel rows, this spill out is the source of the apparent dimerization, as can be seen in Figure 9c. In this image, the partial charge density is shown for energies between 1.37 and 1.47 V. The difference between both grooves is apparent, with the one on the right showing higher charge density, which produces a higher apparent height in the topographic images. The different apparent heights between both grooves are visually interpreted as a dimerization. It is worth noting that the electronic asymmetry of phenyl-TOTA has minimal effect in the geometry of the system. For example, the tilt of the phenyl moiety with respect to the surface normal is smaller than 0.5°.

**Figure 9:**
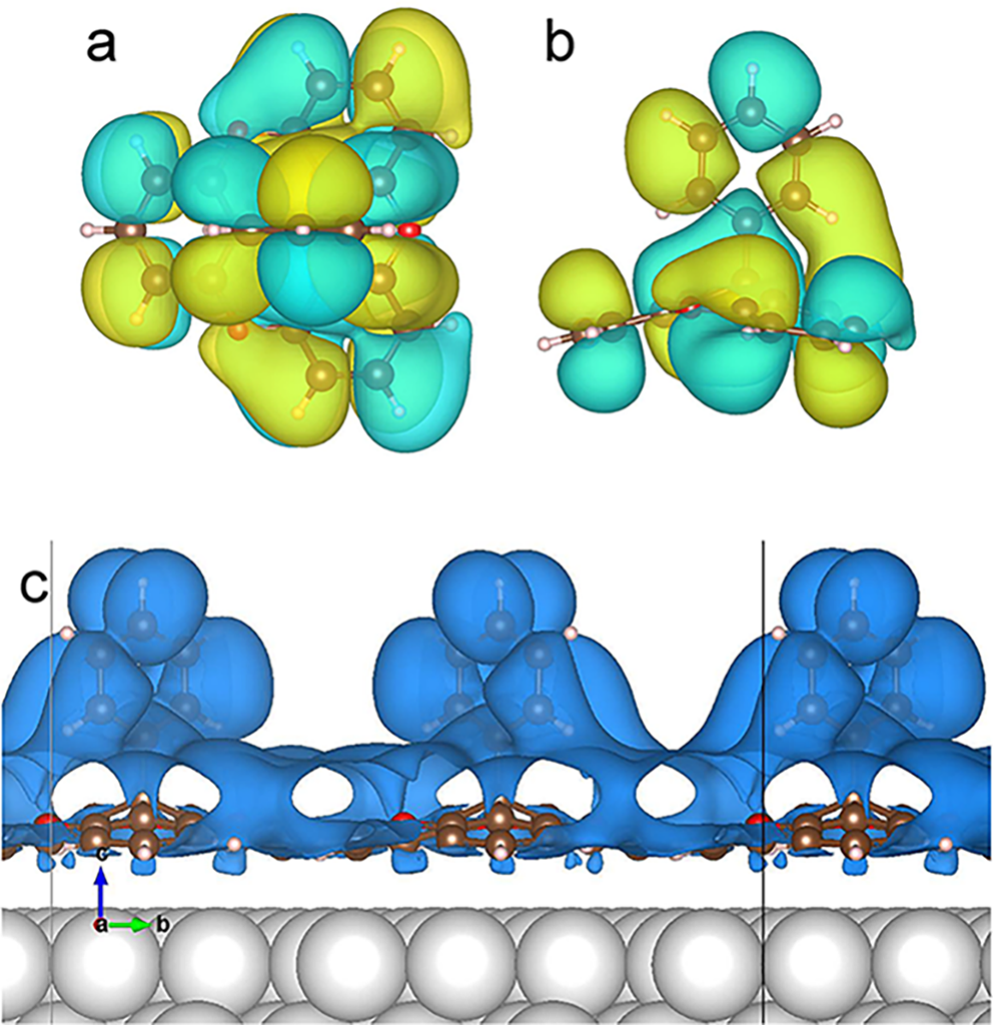
Isodensity contours of the LUMO of the gas-phase phenyl-TOTA viewed from (a) the top and (b) a side. (c) Partial charge density of the structure shown in Figure 7 computed between 1.37 and 1.47 V. The charge density spills out further in the groove on the right side, appearing in the images as an apparent dimerization.

### Discussion

The orientational order of the phenyl moieties, which are separated by a distance of 1 nm, is remarkable and deserves further discussion. We have shown that the orientation of the phenyl ligands can be determined experimentally and it is correctly reproduced by our model. To shed more light on the interactions at play, we considered a larger unit cell, with doubled length in the *a* direction. In this model we can study configurations where phenyl moieties are not parallel in the horizontal direction. Figure 10, presents four characteristic configurations. The energy differences between these configurations can be found in Table 1. The most stable configuration is I, which corresponds to the experimental observation. Configuration II is very close in energy, actually within the estimated error margin of the calculation. The other configurations have significantly higher energies. Next, we consider the energy of the layer of phenyl-TOTA without the surface (*E*_ph–TOTA_ in Table 1). We find that the energy differences are larger, indicating that the orientational order of the phenyl ligands is not due to the interaction through the surface, which is actually detrimental. In the phenyl-TOTA layer, only two contributions are relevant: a direct interaction between phenyl moieties, and an indirect interaction through the TOTA platforms. To elucidate their respective roles we considered a layer of benzene molecules located at the same positions and orientations of the phenyl ligands (*E*_benzene_ in Table 1). Δ*E*_benzene_ comprises the main part of Δ*E*_ph–TOTA_. Therefore, we can conclude that the principal effect is the direct interaction between phenyl moieties. The interaction of the phenyl ligands and the TOTA platform mainly forces the ligands to adopt three possible orientations (pointing to one of the oxygen atoms in the platform). Within this constraint, the preferred orientation is determined by the direct interaction between the phenyl moieties. The binding energy for the benzene layer in configuration I is *E*_B_ = 3.84 meV per molecule. As in the adsorption energy, the vdW part dominates with a value of *E*_B,vdW_ = 3.09 meV.

**Figure 10:**
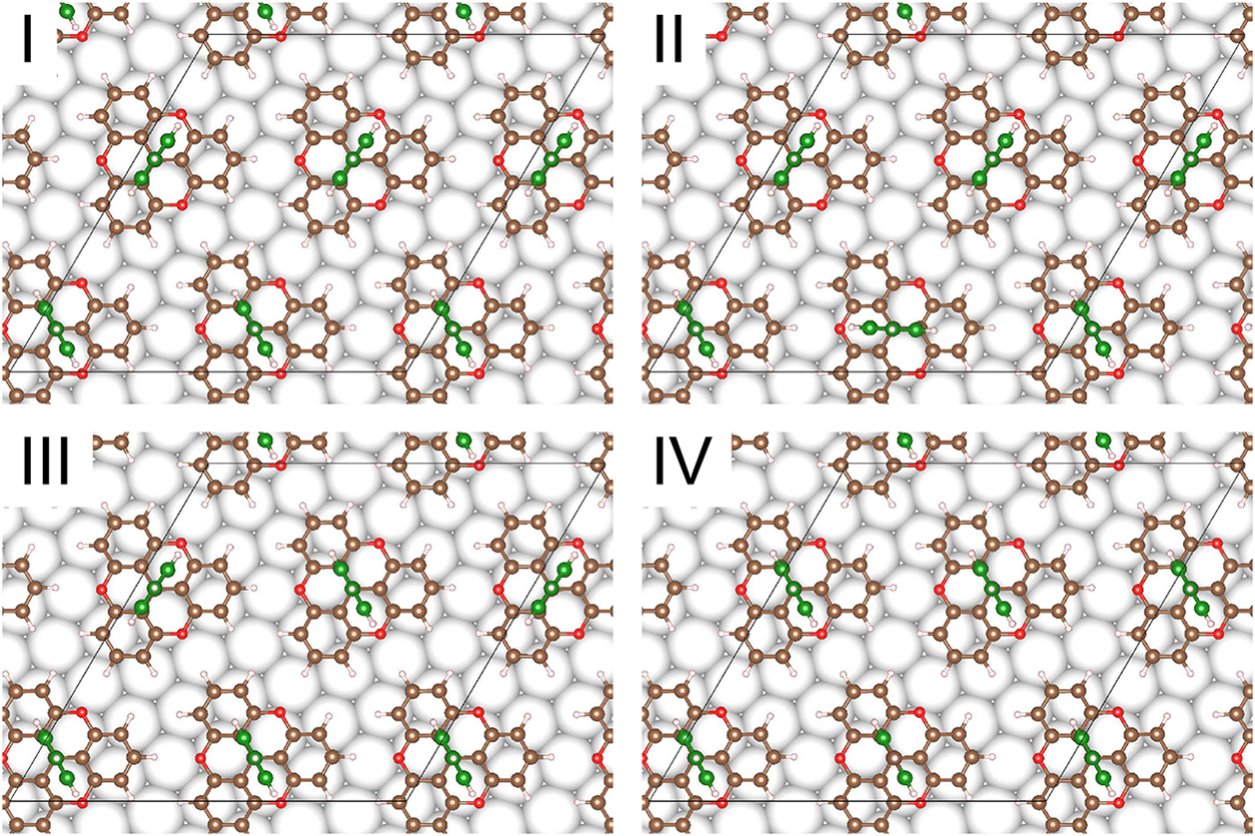
Top views of four configurations in a unit cell comprised of four phenyl-TOTA molecules on Ag(111). White, brown, red, and pink spheres represent Ag, C, O and H atoms, respectively. Green spheres represent C atoms of the phenyl moiety. Black lines show the unit cell.

**Table 1:** Computed Energy Differences of the Different Configurations Shown in Figure 10 with Respect to the Lower Energy One^a^

	Δ*E*_TOTAL_	Δ*E*_ph‑TOTA_	Δ*E*_benzene_
I	0.00	0.00	0.00
II	0.27	1.85	1.79
III	4.91	7.68	6.19
IV	7.40	12.45	8.84

a *E*_TOTAL_ is the total energy of the phenyl-TOTA layer on Ag(111); *E*_ph‑TOTA_ is the energy of the phenyl-TOTA layer without surface; and *E*_benzene_ is the energy of a layer of benzene molecules at the same positions and orientations of the phenyl ligands. All energies are in meV.

We now go a step forward in rationalizing the interaction between the phenyl moieties. In the hexagonal layer of benzene molecules, each molecule directly interacts with 6 neighbors. We simplify this model by studying the interaction within a row in the unit cell of Figure 10. Allowing for the independent rotation of each phenyl around the surface normal we mimic the situation of a phenyl ligand on top of the TOTA platform. The corresponding potential energy surface (PES) as a function of the rotation angles (α, β) is shown in the Supporting Information. We can improve this model by adding the interaction of a phenyl ligand with the TOTA platform, which to first approximation is a penalty equal to 0 when the ligand is pointing to an O atom and maximum when it points in between two O atoms. The resulting PES’ is shown in the Supporting Information. We observe minima around 0 and 60° and around symmetry equivalent orientations. Looking at the ligand orientations in Figure 10, the most stable solution I maximizes the number rows with minimum energy (0, 60°), while only rows with higher energies are present in the least stable solution IV.

Our model qualitatively explains the experimental result, but is based on DFT with the PBE functional and D3 vdW corrections, a relatively simple theoretical approach. In a benchmark of DFT methods versus state-of-the-art CCSD­(T)/CBS calculations, Herman et al.^23^ determined that B3LYP-D3 is a good compromise between accuracy and computational demands. We can check this theoretical approach for our system by considering the benzene–benzene interaction at long distances. For the canonical T-shaped configuration, Czernek et al.^24^ found an interaction energy of 4.47 meV at 0.9 nm. Using B3LYP-D3 we obtained a value of 3.79 meV in reasonable agreement with the CCSD­(T)/CBS result of Czernek et al. We therefore computed PES and PES’ using B3LYP-D3 and found the same qualitative result as for PBE-D3 (see Supporting Information). The main difference between both theoretical methods is the interaction of the phenyl ligand with the TOTA platform, which is three times stronger with B3LYP-D3. This will favor the tendency of the phenyl ligand to align with the O atom of the TOTA platform, but overall the interpretation of the results remains unaltered.

## Conclusions

We observed that phenyl-functionalized trioxatriangulenium (phenyl-TOTA) molecules self-assemble into highly ordered supramolecular networks on Ag(111) and Au(111) surfaces, exhibiting distinct orientational patterns of the phenyl moieties. Using low-temperature STM and DFT calculations with van der Waals corrections, we resolved the orientation of individual phenyl groups and identified an intriguing dimer-like contrast effect on Ag(111), which is reproduced in the simulated STM images. This apparent dimerization arises from an asymmetry of the phenyl wave function, induced by intramolecular hydrogen bonding between the phenyl ligand and an oxygen atom of the TOTA platform.

Our theoretical analysis reveals that the orientational order of the phenyl groups is governed primarily by direct long-range interactions between phenyl moieties, rather than substrate-mediated effects. The TOTA platform constrains the phenyl ligands to three symmetry-equivalent orientations, and the final arrangement is determined by minimizing the intermolecular interaction energy between neighboring phenyl groups. This interplay between intramolecular constraints and intermolecular interactions leads to the emergence of long-range orientational order and surface-induced chirality, as observed in the experiments.

These findings provide new insights into the design principles of functional molecular assemblies on surfaces, highlighting the role of subtle electronic and long-range effects in directing supramolecular organization. The phenyl-TOTA molecule serves as a model for studying aromatic–aromatic interactions in a controlled fashion and may open an avenue for engineering surface-confined molecular architectures that enable systematic exploration of long-range molecular interactions.

## Methods

### Experimental Details

Experiments were carried out with a STM operated at ≈4.6 K in ultrahigh vacuum. Ag(111) surfaces were prepared by cycles of Ar sputtering (ion energy 1.5 keV) and annealing to 500 °C. Phenyl-TOTA molecules were sublimated from a crucible heated to ≈100 °C onto the substrate at ambient temperature. STM tips were electrochemically etched from W wire and ion bombarded *in vacuo*. STM topographs were recorded at a fixed current *I* = 10 pA unless otherwise indicated. The bias voltage *V* was applied to the sample. For spectroscopy of the differential conductance d*I*/d*V*, a sinusoidal modulation with 10 mV_RMS_ amplitude and a frequency between 410 and 685 Hz was added to the bias.

### Computational Details

Density functional theory calculations were performed using the VASP code.^31^ The projector augmented-wave method^32^ was used to treat core electrons. Wave functions were expanded using a plane wave basis set with an energy cutoff of 500 eV. Unless noted, the PBE-D3 method was used for all calculations, consisting in the combination of the PBE exchange and correlation functional^33^ complemented with the D3 method^34,35^ to treat van der Waals interactions. Additional calculations were performed using the B3LYP-D3 method, which combines the hybrid B3LYP^36^ functional with the D3 method.

To simulate the experimentally determined periodicity of the molecular layer, the slab method was used with 4 layers of Ag and an in-plane unit cell determined by $\left(\begin{array}{ll} 4 & 2\\ -1 & 6 \end{array}\right)$ , accommodating two molecules. A (4 × 2 × 1) *k*-grid was used to sample the Brillouin zone. The electronic structure was converged with a tolerance of 10^–7^, while geometries of all atoms except the two bottom Ag layers were relaxed until forces were smaller than 0.01 eV/Å.

Adsorption energies *per* adsorbed molecule were calculated using

$$E_{\mathrm{ad}}\left[n\cdot \left(\mathrm{ph}‐\mathrm{TOTA}\right)@\mathrm{Ag}\left(111\right)\right]=[n\cdot E\left[\mathrm{ph}‐\mathrm{TOTA}\right]+E\left[\mathrm{Ag}\left(111\right)\right]-E\left[n\cdot \mathrm{ph}‐\mathrm{TOTA}@\mathrm{Ag}\left(111\right)\right]/n$$

where *n* is the number of adsorbed molecules, *E*[ph-TOTA] is the energy of one molecule, *E*[Ag­(111)] is the energy of the Ag(111) surface, and *E*[*n* · ph-TOTA@Ag(111)] is the energy of the full system. When computing the energies *E* denotes the total energy, *E*_vdW_ denotes the van der Waals part, and *E*_PBE_ = *E* – *E*_vdW_ denotes all contributions except the vdW part.

STM images were simulated within the Tersoff-Hamann approximation^37^ using the method of Bocquet et al.^38^ as implemented in the STMpw code.^39^ Atomic and density plots were generated using the VESTA program.^40^

## Supplementary Material



## Footnotes

1 When a bulky ligand with lateral dimensions exceeded those of the TOTA platform were used, other patterns have been observed.^13^

2 We use the binding energy determined with STM, which is shifted by approximately 4 meV compared to photoelectron spectroscopy data because of the impact of the STM tip on the local potential.^27,28^

## Notes

Additional STM topographs and spectroscopy data as well as further results from DFT calculations (PDF)
