# Supplementary material for: Orientational Order of Phenyl Rotors on Triangular Platforms on Ag and Au(111)
Source: ACS Nano. 2025 Oct 27;19(44):38773–80. doi: 10.1021/acsnano.5c14953 (PMC12613842; doi:10.1021/acsnano.5c14953)
Supplement: Supplementary file 1 [file nn5c14953_si_001.pdf]

Supplementary Information for
Orientational Order of Phenyl Rotors on Triangular Platforms on Ag and Au(111)

Behzad Mortezapour,[1] Sebastian Hamer,[2] Rainer Herges,[2] Roberto Robles,[3,*] and Richard Berndt[1,†]

[1]*Institut für Experimentelle und Angewandte Physik, Christian-Albrechts-Universität zu Kiel, 24098 Kiel, Germany*
[2]*Otto-Diels-Institut für Organische Chemie, Christian-Albrechts-Universität zu Kiel, 24098 Kiel, Germany*
[3]*Centro de Física de Materiales CFM/MPC (CSIC-UPV/EHU), 20018 Donostia-San Sebastián, Spain*
(Dated: October 24, 2025)

A. Imaging on Ag(111) at Negative Voltages

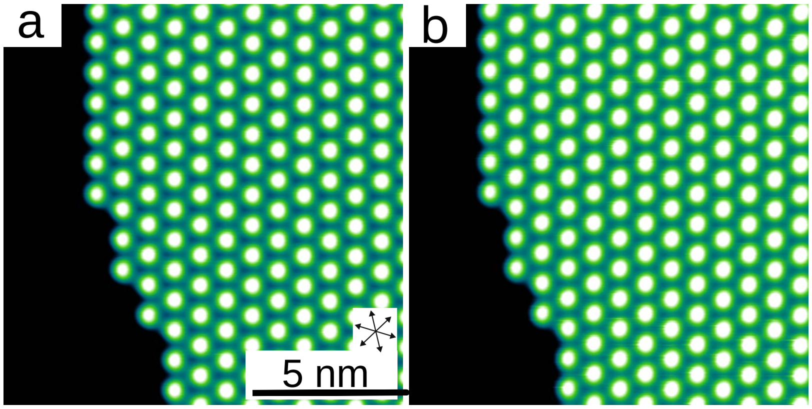

FIG. S1. Topographs of a molecular island obtained at (a) $V = -0.5$ and (b) $-2.5\,\mathrm{V}$. The image contrast is hardly voltage dependent and resembles images observed at low positive voltages.

B. Apparent Height of phenyl-TOTA on Ag(111)

Consistent with the dI/dV spectra, there is little variation of the apparent height (Table SI) at negative and small positive voltages and a steep increase of the height at 2 V and above.

V (V)	−0.5	−1.0	−1.5	−2.0	−2.5	0.5	1.0	1.5	2.0	2.5
Height (pm)	355	345	340	340	320	335	360	375	400	485

TABLE SI. The apparent height of phenyl-TOTA relative to the substrate recorded with $I = 10\,\mathrm{pA}$. The uncertaincies are $\pm 5\,\mathrm{pm}$ except at $-0.5\,\mathrm{V}$, where the margin is $\pm 10\,\mathrm{pm}$.

* roberto.robles@csic.es
† berndt@physik.uni-kiel.de

C. Perturbation by the Tip at Elevated Voltage

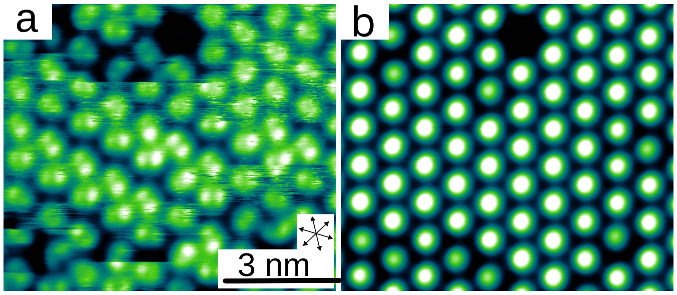

FIG. S2. (a) Topograph of the surface area shown in Fig. 2 of the main text, recorded at $V = 2.6\,\mathrm{V}$. At this voltage, the tunneling current is unstable and leads to abrupt changes and streaks in the topograph. (b) Topograph recorded subsequently under non-perturbative conditions, $V = 0.3\,\mathrm{V}$. Some molecules appear approximately 30 pm lower prior to the manipulation. This effect may be due to hydrogen atoms missing from the phenyl subunit.

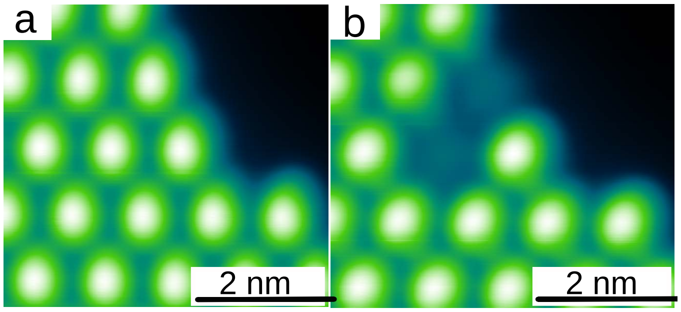

FIG. S3. Removal of the phenyl ligands from two phenyl-TOTA molecules. (a) Image of the area before manipulation. (b) Image recorded after applying a voltage pulse to two molecules close to the image center. During the 20 ms pulses, the voltage was rapidly raised from 0.15 to 3.7 V.

D. Image of a Ag(111) Step

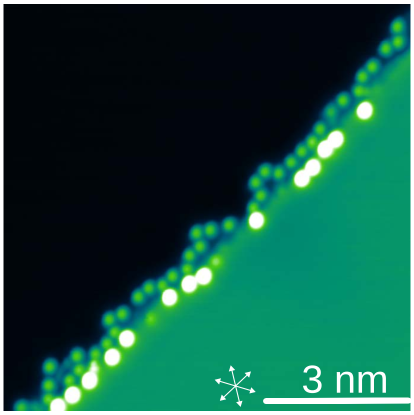

FIG. S4. Topograph of a typical monatomic step of the Ag(111) substrate. While the majority of protrusions observed at the step on both terraces are consisten with being phenyl-TOTA, some features appear lower, possibly due to fragments or other impurities.

E. dI/dV Spectra on Au(111)

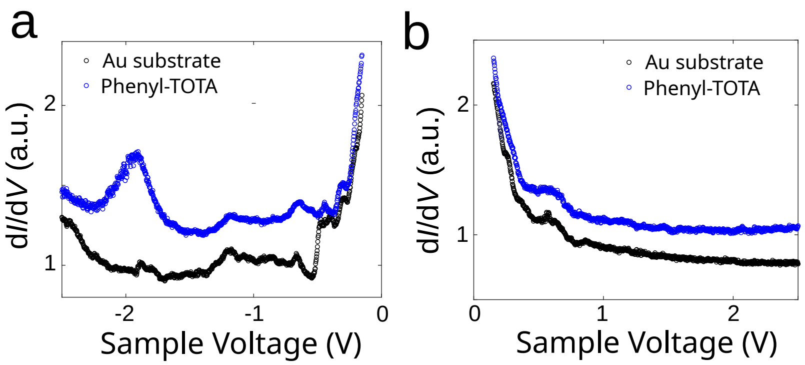

FIG. S5. Constant-current ($I = 50\,\mathrm{pA}$) dI/dV spectra of phenyl-TOTA on Au(111) (blue lines) and of the substrate (black lines). (a) Negative voltages. (b) Positive voltages.

F. Determination of the Adsorption Sites of an Isolated phenyl-TOTA molecule

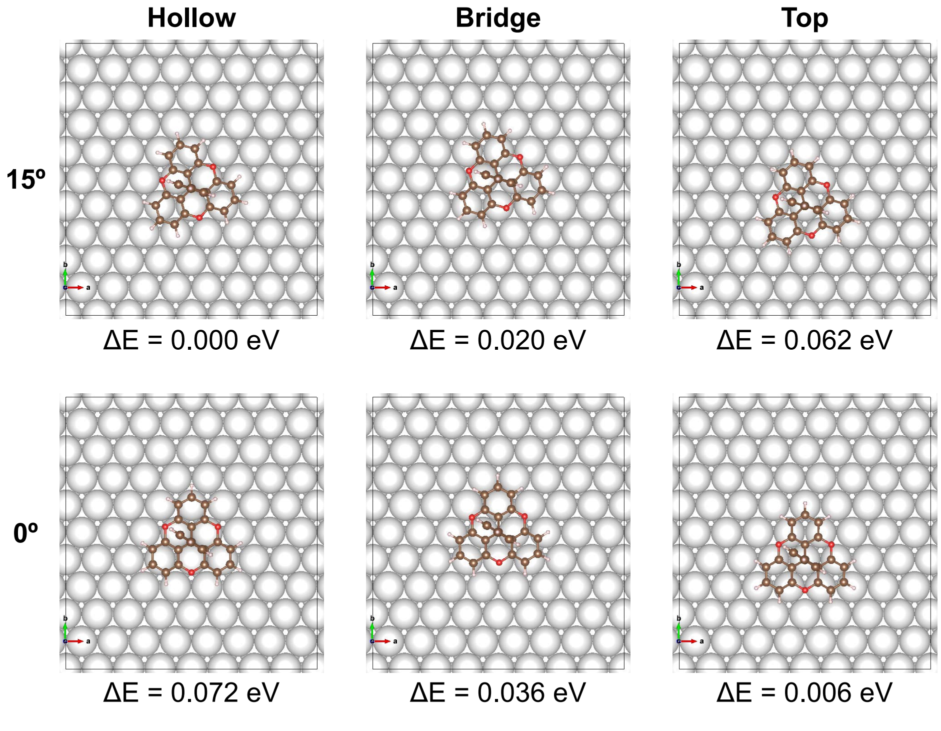

FIG. S6. Six geometries of an isolated phenyl-TOTA molecule on Ag(111). 0° and 15° rotations with respect to the $(1\bar{1}0)$ direction are considered. The center of the molecule is placed at hollow, bridge or top positions. Gray, brown, red, and pink spheres represent Ag, C, O and H atoms, respectively. Black lines show the unit cell. The energy differences of all configurations are indicated below each panel.

To determine the most stable configuration of phenyl-TOTA molecules on Ag(111) we considered a rectangular (8×5) unit cell with just one isolated molecule. The experiments indicate a ≈ 15° rotation of the molecules with respect to the $(1\bar{1}0)$ densely packed direction on the (111) surface. We then considered 0° and 15° rotations of the molecules and hollow, bridge and top positions for the center of the molecule (Figure S6). The most stable configuration corresponds

to a molecule rotated by 15° at the hollow position. We used this geometry for the construction of the unit cells of the monolayer.

G. Simulation of Different Ligand Orientations

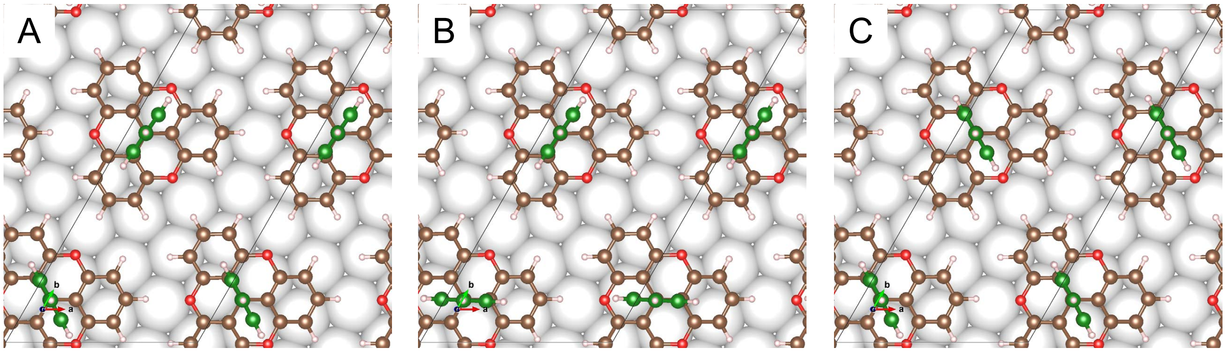

FIG. S7. Three different orientations of the phenyl ligands in the cell containing two inequivalent phenyl-TOTA molecules. Configuration A is discussed in the main text. White, brown, red, and pink spheres represent Ag, C, O and H atoms, respectively. Green spheres represent C atoms of the phenyl moiety. Black lines show the unit cell.

We considered different orientations of the phenyl ligands, which are shown in Figure S7. The corresponding energy differences and adsorption energies are shown in Table SII. The energy differences between the configurations are small, of the order of meV. However, as seen in Figure S8, the simulated STM images at $V = 1.6$ V are quite different and only configuration A is compatible with the experimental observations.

The properties of this system are mainly governed by vdW interactions. Many approaches to include them are suggested in the literature. We have tested five of them: two are semiempirical pairwise corrections (PBE-D3 [1, 2] and PBE+vdW^{surf} [3]), while the other three are nonlocal vdW-DF functionals in the spirit of Dion *et al* [4]. Regardless of the approach, configuration A is found to be the ground state, while the other two configurations are within few meV. The variation of the adsorption energies is wider, going from 1.6 to 2.2 eV per molecule. The van der Waals part of the adsorption energy dominates in all cases. The distance between the molecule and the surface is similar for all methods, varying from 2.7 to 2.9 Å. For reference, the distance for the PBE functional, without including any vdW correction, is of 3.4 Å. In summary, the same qualitative picture emerges from all methods and the determination of the experimental configuration is consistent for all of them.

	**A**			**B**			**C**			
	ΔE	E_{ad}	$E_{\mathrm{ad,vdW}}$	ΔE	E_{ad}	$E_{\mathrm{ad,vdW}}$	ΔE	E_{ad}	$E_{\mathrm{ad,vdW}}$	d
PBE-D3 [1, 2]	0.00	2.238	2.584	2.47	2.237	2.588	3.69	2.236	2.594	2.8
PBE+vdW^{surf}[3]	0.00	2.140	2.735	0.40	2.139	2.760	3.72	2.138	2.753	2.7
Hamada [5]	0.00	1.649	2.886	3.33	1.647	2.892	3.18	1.647	2.893	2.9
CX [6]	0.00	1.954	1.759	3.65	1.952	1.752	3.93	1.952	1.771	2.8
optB86b [7]	0.00	2.114	3.777	2.97	2.113	3.783	3.11	2.113	3.792	2.9

TABLE SII. Energy differences ΔE (in meV), adsorption energies E_{ad} and $E_{\mathrm{ad,vdW}}$ (in eV per molecule) and distances d (in Å) between the molecule and the Ag(111) surface. They are shown for the three configurations in figure S7 and for five different theoretical approaches.

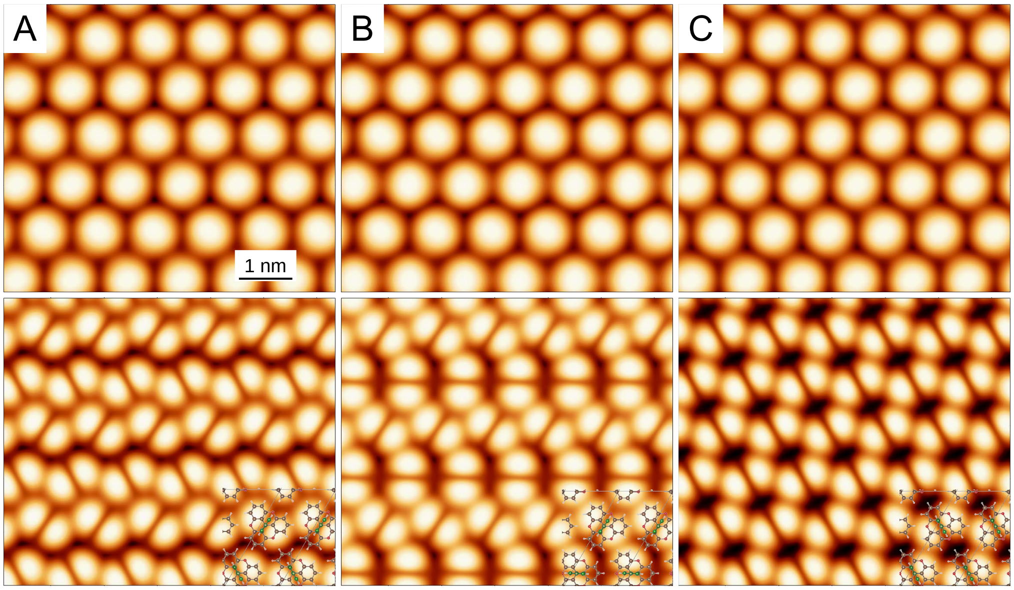

FIG. S8. Constant-current STM images simulated for the three configurations of Figure S7. The first row was computed for $V = 0.5\,\mathrm{V}$. The images are essentially featureless and independent of the phenyl orientation. The second row at $V = 1.6$ V captures the LUMO and reveals the different orientations of the phenyl moieties. The molecular structure is overlaid in the bottom images.

H. Chain of Benzene Molecules

We studied the direct interaction between phenyl moieties using the model shown in Figure S9a. It consists of a chain of benzene molecules at the same distance as the phenyl moieties in the cell of Fig. S7. The benzenes are constrained to rotate in a vertical axis mimicking the phenyl ligands of phenyl-TOTA. We considered two inequivalent molecules rotated by angles α and β. We varied these angles from 0° to 120° in 10° intervals and computed the energy differences to obtain the PES as a function of (α, β) (Figure S9b). The minimum energy is found at $(20°, 50°)$ and corresponds to a structure resembling the global minimum of a benzene dimer, namely a tilted T-shaped configuration [8]. There is ample region with an energy within 1 meV of the minimum. The maximum energy configuration is 5.04 meV higher in energy and corresponds to $(90°, 90°)$, with the benzenes parallel and facing each other.

The model can be improved by adding the energy of rotating a phenyl ligand on top of the TOTA platform. This energy is minimal for multiples of 60°, when the phenyl ligand is pointing to an oxygen atom, and maximum for 30°, when the ligand is parallel to one side of the TOTA platform. We add to the PES the values for each angle going from 0 meV for the minimum to 1.39 meV for the maximum, obtaining the modified PES' in Figure S9c. The minima are around $(0°, 60°)$ and symmetry equivalent combinations $(60°, 0°)$, $(0°, 120°)$ and $(120°, 0°)$. Using the results in PES' we can rationalize the most stable configuration in the layer of phenyl-TOTA molecules shown in Figure S7A as the one that maximizes the number of rows with the minimum energy configuration. Conversely, for configuration C none of the rows corresponds to a orientation of phenyl ligands similar to the minimum energy one.

The same analysis was done for the B3LYP-D3 functional, which according to Herman *et al.* [9] is good choice for studying benzene–benzene interactions. The results are shown in Figure S9d and e. For the PES, a similar shape is obtained. Now the maximum at $(90°, 90°)$ is 5.79 meV higher than the minimum at $(10°, 70°)$. This value is not far from the 5.04 meV value found for PBE-D3. The fact that the PES's computed with these two methods are similar reflects a comparable long-range interaction due to the van der Waals forces. However, the interaction with the TOTA platform is stronger within B3LYP-D3 (between 0 and 4.36 meV) compared to 1.39 meV for PBE-D3. As can be seen in PES' (Figure S9e), this stronger interaction further stabilizes the configurations at $(0°, 60°)$ and in general configurations where α and β are multiples of 60°. This fact reinforces the agreement with the experimental observations, where the phenyl ligands are only observed with angles multiple of 60°. In conclusion, the intramolecular interaction of the phenyl moieties on the TOTA platform is the major difference between the PBE-D3 and the B3LYP-D3 methods. Overall, the same qualitative interpretation of the experimental results is achieved with both theoretical methods.

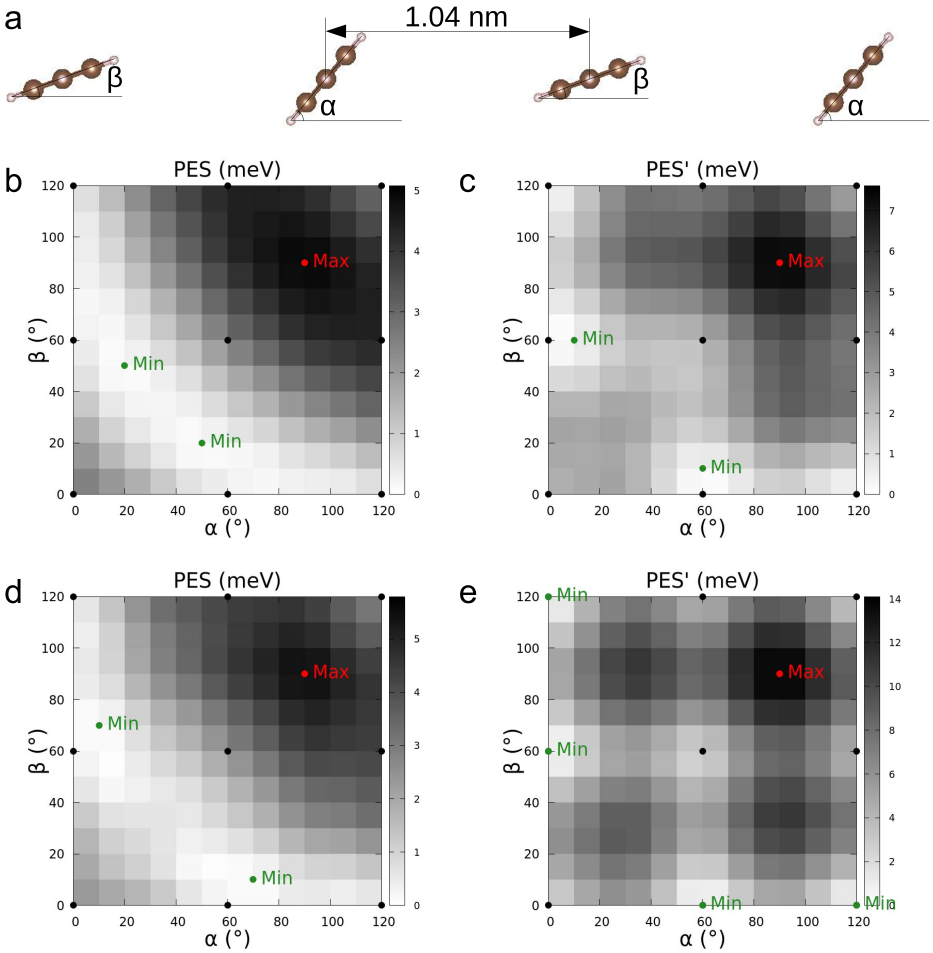

FIG. S9. (a) Schematic top view of a chain of benzene molecules with two inequivalent molecules rotated by angles α and β, respectively. The molecular centers are separated by 1.04 nm. The model simulates the direct long-range interaction of the phenyl ligands on the TOTA platform. (b) PBE-D3 potential energy surface (PES) of the model in (a) as a function of the angles (α, β). Minimum (maximum) energy points are marked in green (red). Black dots indicate multiples of 60°. (c) PBE-D3 PES' including the interaction of a phenyl ligand in the TOTA platform for the given angle. (d–e) PES as (b) and (c), but calculated with the B3LYP-D3 functional and vdW correction.

[1] S. Grimme, J. Antony, S. Ehrlich, and H. Krieg, A Consistent and Accurate Ab Initio Parametrization of Density Functional Dispersion Correction (DFT-D) for the 94 Elements H–Pu, J. Chem. Phys. **132**, 154104 (2010).

[2] S. Grimme, S. Ehrlich, and L. Goerigk, Effect of the Damping Function in Dispersion Corrected Density Functional Theory, J. Comp. Chem. **32**, 1456 (2011).

[3] V. G. Ruiz, W. Liu, and A. Tkatchenko, Density-Functional Theory with Screened van der Waals Interactions Applied to Atomic and Molecular Adsorbates on Close-Packed and Non-Close-Packed Surfaces, Phys. Rev. B **93**, 035118 (2016).

[4] M. Dion, H. Rydberg, E. Schröder, D. C. Langreth, and B. I. Lundqvist, Van der Waals Density Functional for General Geometries, Phys. Rev. Lett. **92**, 246401 (2004).

[5] I. Hamada, Van der Waals Density Functional Made Accurate, Phys. Rev. B **89**, 121103 (2014).

[6] K. Berland and P. Hyldgaard, Exchange Functional that Tests the Robustness of the Plasmon Description of the van der Waals Density Functional, Phys. Rev. B **89**, 035412 (2014).

[7] J. Klimeš, D. R. Bowler, and A. Michaelides, Van der Waals Density Functionals Applied to Solids, Phys. Rev. B **83**, 195131 (2011).

[8] J. Czernek and J. Brus, Revisiting the Most Stable Structures of the Benzene Dimer, Int. J. Mol. Sci. **25**, 8272 (2024).

[9] K. M. Herman, E. Aprà, and S. S. Xantheas, A Critical Comparison of CH$\cdots\pi$ versus $\pi\cdots\pi$ Interactions in the Benzene Dimer: Obtaining Benchmarks at the CCSD(T) Level and Assessing the Accuracy of Lower Scaling Methods, Phys. Chem. Chem. Phys. **25**, 4824 (2023).
